# Optimization and Evaluation of Desloratadine Oral Strip: An Innovation in Paediatric Medication

**DOI:** 10.1155/2013/395681

**Published:** 2013-10-21

**Authors:** Harmanpreet Singh, Mandeep Kaur, Hitesh Verma

**Affiliations:** School of Pharmaceutical Sciences, Lovely Faculty of Applied Medical Sciences, Lovely Professional University, Phagwara 144411, Punjab, India

## Abstract

Patients, especially children, are the most difficult to treat in all groups of population mainly because they can not swallow the solid dosage form. Due to this reason they are often prescribed liquid dosage forms. But these formulations have their own disadvantages (lack of dose accuracy during administration, spitting by children, spillage, lack of stability, difficulty in transportation, etc.). Oral strip technology is one such technology to surpass these disadvantages. Desloratadine, a descarboethoxy derivative of loratadine, is a second generation antihistaminic drug approved for usage in allergic rhinitis among paediatric population and is available in markets as suspension. An attempt has been made to design and optimize the oral strip containing desloratadine as an active ingredient. Oral strip was optimized with the help of optimal experimental design using polymer concentration, plasticizer type, and plasticizer concentration as independent variables. Prepared oral strips were evaluated for physicochemical parameter, mechanical strength parameters, disintegration time, dissolution, surface pH, and moisture sorption tendency. Optimized formulation was further evaluated by scanning electron microscopy, moisture content, and histological alteration in oral mucosa. Accelerated stability studies were also carried out for optimized formulations. Results were analysed with the help of various statistical tools at *P* < 0.05 and *P* < 0.01.

## 1. Introduction

Allergic rhinitis (AR) is allergen driven immune mediated disorder characterized by nasal congestion, nasal pruritus, rhinorrhea, and sneezing. Traditionally, AR is classified as Seasonal or Perennial. According to Allergic Rhinitis and its Impact on Asthma (ARIA) guidelines, AR can be broadly classified into intermittent (≤4 days/week or ≤4 weeks/year) or persistent (>4 days/week or >4 weeks/year). Exhaustive literature survey shows that about 40% of the children are suffering from AR, but still these figures seem conservative as AR is often confused with common cold by physicians [1, 2]. Desloratadine (DSL), a descarboethoxy derivative of loratadine, is a second generation anti histaminic drug approved by FDA for paediatric usage. It is given as dose of 1.25 mg for children aged 2–5 years, that is, preschool children and 2.5 mg for children aged 6–11 years [3].

Paediatric population, because of its wide assortment, is the most difficult to treat among all age groups because they often meet difficulty in swallowing of solid dosage forms. Due to this reason, they are usually prescribed with solution, emulsion, suspensions, and so forth, but these dosage forms have their own limitations like lack of dosage accuracy, spillage chances, difficulty in transportation, and above all frequent spitting out by paediatric patients [4]. 

Oral strip technology (OST) is an innovative drug delivery technology which can provide solution for the disadvantages of liquid dosage form and bring together the advantages of solid dosage form. In addition, due to its flexible nature it gives durability to the formulation [5]. Oral strip is a unique, thin postage stamp sized dosage form required to be placed on the tongue where it will disintegrate instantaneously by absorbing saliva without the need of water and will turn into a suspension or a solution which will be easily swallowed by the child. There are very less chances of spitting out because the strip will disintegrate in few seconds and will adhere to oral mucosa [6–8].

In addition to Active Pharmaceutical Ingredient (API), major components of OS are film forming polymer and plasticizer, which impart desired shape and elasticity to oral strip (OS). Specific examples of film forming polymers that have been used for OS involve Pullulan, Hydroxy Propyl Methyl Cellulose (HPMC), Hydroxy Ethyl Cellulose (HEC), Povidone K-90, Xanthan gum, Tragacanth gum, Guar gum, Acacia gum, Arabic gum, methyl methacrylic copolymer, carboxyvinyl copolymer, or combinations thereof. Among plasticizers various noteworthy examples are glycerol, propylene glycol, polyethylene glycol (PEG), and so forth [9–14].

DSL is an ideal drug candidate for OST because of its low dose and its high efficiency in treating AR among paediatrics and adults. It is known to be rapidly absorbed via oral administration. Therefore, the aim of present work was to design and characterize OS of DSL for paediatrics using the blend of two polymers using optimal experimental design. Independent variables were chosen, namely, polymer type, polymer ratio, plasticizer type, and plasticizer ratio. Effect of independent variables was evaluated on various film properties, namely, mechanical strength (tensile strength and elasticity), disintegration time, and dissolution time. Various other properties of film were also evaluated, namely, weight variation, percent moisture uptake, film thickness, and water content present in film.

## 2. Materials and Methods

### 2.1. Materials

DSL was obtained as a gift sample from Sun Pharmaceuticals (India). Maltodextrin (MDX) having dextrose equivalent value (DE) 13 to 17 was procured from Loba Chemie (India). HPMC K4M and HPMC E-5 were obtained from Colorcon (Italy) and Central drug house Pvt. Ltd. (India), respectively. Poloxamers 407 (P407) and 188 (P188) were obtained as a gift sample from BASF (Germany). Glycerol and PEG-400 were procured from Qualikems fine chem Pvt. Ltd. (India). All other chemicals used were of analytical grades. 

### 2.2. UV Spectroscopic Method

Calibration curve of DSL was constructed in phosphate buffer saline (PBS) at pH 6.8 and 0.1 hydrochloric acid (HCl) at pH 1.2. Stock solutions were prepared by accurately weighing 10 mg of DSL which was transferring it to precalibrated 25 mL volumetric flasks, and volume was made up to the mark using suitable media. Serial dilutions were prepared from stock solutions, and absorbance was measured using UV spectrophotometer (Systronics 2203, India) at 242 nm. No excipient mediated fluctuation in *λ*_max⁡_ was observed. Beer Lambert law was followed over the range of 5–30 *μ*g/mL with a regression coefficient (*R* ^2^) value of 0.999. 

### 2.3. Saturated Solubility Determination

Saturated solubility of DSL was determined in PBS alone and in presence of P188 and P407 (0.5% w/v, 2% w/v, and 5% w/v). A known excess amount of DSL (100 mg) was mixed with 5 mL of PBS in a glass vial followed by shaking on a thermostatically controlled magnetic stirrer (Rajendra Electrical Industries Ltd, India) at 37°C ± 0.5°C for 72 hours. Resultant suspension was filtered through 0.45 *μ*m Millipore filter. Filtrate was diluted appropriately with PBS and analysed by using UV spectrophotometer at 242 nm. All the measurements were performed in triplicate. Similar procedure was adopted in case of solution containing different concentration of P188 and P407 in PBS [13].

### 2.4. Preparation of Film

**Figure 1:**
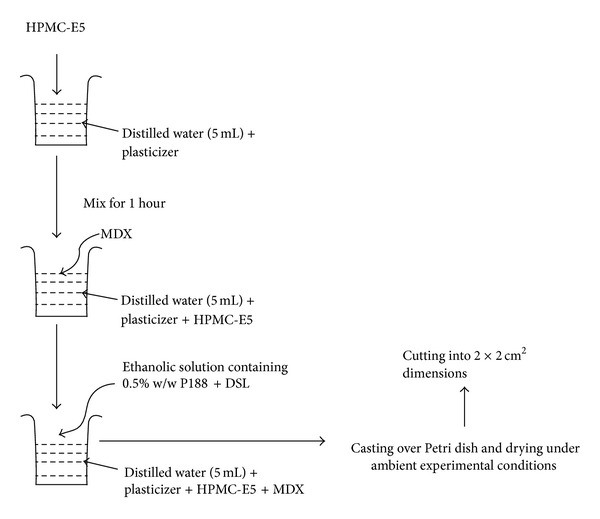
Preparation of OS of DSL.

OS of DSL was prepared by adopting solvent casting methodology. Based on the exhaustive literature survey MDX and HPMC E-5 were taken in three different ratios, namely, 9 : 1, 7 : 3, and 5 : 5 with PEG 400, and glycerol as plasticizer in 15%, 20%, and 25% (w/w) concentration. Procedure of film preparation is briefly described in Figure 1.

### 2.5. Optimal Experimental Design

Three independent variables were selected in the study, namely, HPMC E-5/MDX (5 : 5, 7 : 3, and 9 : 1), type of plasticizer (PEG 400 or glycerol), and concentration of plasticizer (15%, 20%, and 25% w/w). Dependent variables chosen were mechanical properties of film (tensile strength and percent elongation), disintegration time, and dissolution time. Optimal experimental design (Table 1) was prepared and statistically evaluated by using Design-Expert version 8 software.

### 2.6. Evaluation of OS of DSL

#### 2.6.1. Weight of Film and Thickness

Prepared film was cut into 2 × 2 cm^2^ area. Weight of each of film was recorded. Thickness of film (*n* = 3) was calculated by using micrometre (Mitutoyo corporation, Japan) having a least count of 0.001 mm at three different positions.

#### 2.6.2. Content Uniformity of Film

A 2 × 2 cm^2^ film was dissolved in 10 mL of PBS and was filtered through 0.45 *μ*m Millipore filter, and after making suitable dilutions was analysed by UV spectrophotometer at 242 nm (*n* = 6).

#### 2.6.3. Mechanical Properties of Film

Mechanical properties of film (tensile strength and percent elongation) were evaluated using Instron universal testing instrument (ADMET, 7601 expert series, USA) equipped with 2 kg load cell. The film strip (2 × 2 cm^2^) was held in between the two clamps. Both clamps were positioned at the distance of 5 cm. Film was pulled by the upper clamp at the rate of 10 cm min^−1^. 

Tensile strength or stress at rupture was calculated as a ratio of maximum load applied to the original cross-sectional area of film. It was expressed as Mega Pascal (MPa). Percent elongation was computed by using the following equation:

(1) E(%)=[Increase in distance between the grips(cm)  + Original distance(cm)]×100,

where *E* is percent elongation of film.

#### 2.6.4. Moisture Uptake

Different film formulations were placed in desiccator for 24 hours for complete drying and were weighed individually, followed by exposure to 75% RH at room temperature for 7 days. At different time intervals film formulations were weighed and increase in weight was calculated as a function of moisture uptake. All the measurements were recorded as a replicate of six (*n* = 6).

#### 2.6.5. *In Vitro* Disintegration Time

*In vitro* disintegration time of all the formulations was analyzed by adopting visual method [14, 15]. Film strip was placed in a Petri dish (internal diameter 5 cm) containing 10 mL of PBS at 37°C. Petri dish was swirled at every 10 seconds. Disintegration time was considered as a time when film starts to disintegrate. All the measurements were done in a replicate of six (*n* = 6).

#### 2.6.6. Surface pH of Film

Film (2 × 2 cm^2^) was placed in a Petri dish (6.5 cm, internal diameter), moistened with distilled water, and kept for 1 minute. Electrode of calibrated pH meter (Systronics, India) was bought in contact with the surface of film and allowed to equilibrate for 1 minute [16]. All the measurement were done in replicate of six (*n* = 6).

#### 2.6.7. *In Vitro* Dissolution Testing

Release of DSL was studied using USP II (paddle) dissolution apparatus (Labindia, India). An in-house method was adopted so as to mimic the *in vivo* conditions (adhesion to oral mucosa) and to prevent the floating of OS of DSL. Film was fixed to the bottom of a 250 mL flat bottom beaker in which 120 mL of PBS is at 37°C ± 0.5°C. Rotational speed of paddle was adjusted to 50 rpm. Method was developed as well as validated as per ICH guidelines. Aliquot of 3 mL was withdrawn at different time intervals (1, 2, 4, 8, 16, and 32 minutes). Withdrawn samples were filtered and subsequently analysed by UV spectrophotometer [15].

#### 2.6.8. Determination of Moisture Content in OS of DSL

Optimized formulations (F4, F12, F17, and F21) were stored in desiccator for 7 days as well as exposed to 60% RH for 7 days at room temperature. Percent water content was determined with the help of Karl Fisher titration. Karl Fischer titrator was firstly calibrated using anhydrous methanol followed by analysis of water content which was done by taking 100 mg of film. Water content was determined as a function of amount of iodine consumed in the reaction [15].

#### 2.6.9. Scanning Electron Microscopy (SEM) of Film

F12 formulation was prepared both in presence and in absence of 0.5% w/v poloxamer P188 and was observed and compared with the help of scanning electron microscope, so as to evaluate the role of poloxamer P188 in formulation.

#### 2.6.10. Histological Studies

Optimized samples of film were exposed to moisten freshly excised porcine oral mucosal tissues (upper surface of tongue, hard palate, and soft palate) for 2 hours. Tissue and film were moistened with 2 mL of PBS. After exposure to the film, tissue was thoroughly cleaned with PBS and was observed under optical microscope after suitable staining. Tissues were observed for any change with respect to their normal architecture (taken as control).

#### 2.6.11. Accelerated Stability Studies

Optimized OS was wrapped in aluminium pouch and was sealed. It was stored at 40°C and 75% RH for a period of 3 months. Films were evaluated for their drug content, surface pH, *in vitro* disintegration time, and *in vitro* dissolution time.

## 3. Results and Discussion

Formulation of OS requires many aspects to be considered simultaneously. Drug chosen to be incorporated in OS should have low dose (upto 3% w/w dry weight of the formulation). Since DSL has low dose (1.25 mg), it appears to be a good candidate to incorporate into film dosage form. Moreover, OS of DSL will help to get par from many problems associated with the paediatrics medication [8, 17].

Polymers of OS constitute another important aspect of OS, since they constitute at least 45% w/w of OS. Therefore, criterion for selection of polymer and their concentration to be used is very important. Polymers of OS not only provide desired mechanical properties to the film (shape and strength) but also modulate the release of drug from the formulation. MDXs comprise a mixture of oligosaccharides produced by hydrolysis of starch. They are classified based on their Dextrose Equivalence (DE) value which inversely relates to molecular weight of MDX. Present study involves the usage of MDX of low molecular weight (DE = 13 to 17) because it will impart high solubility to film formulation. HPMC is known for its film forming ability; therefore, an attempt has been made to use both MDX and HPMC in combination. This attempt will impart good solubility to OS due to the presence of the former and will provide good mechanical strength due to the presence of the latter [17, 18].

HPMC was also characterized to study the effect of grades. Two grades, namely, E5 and K4M, were characterized; it was found that the latter decreases the release of drug from OS to statistically significant extent (*P* < 0.05). It is because HPMC-K4M has higher viscosity, after getting hydrated from the surrounding media, it forms gelatinous layer which will act as a diffusional barrier for the release of drug [8]. Hence, HPMC-E5 was selected to form OS.

### 3.1. Saturated Solubility Determination

**Figure 2:**
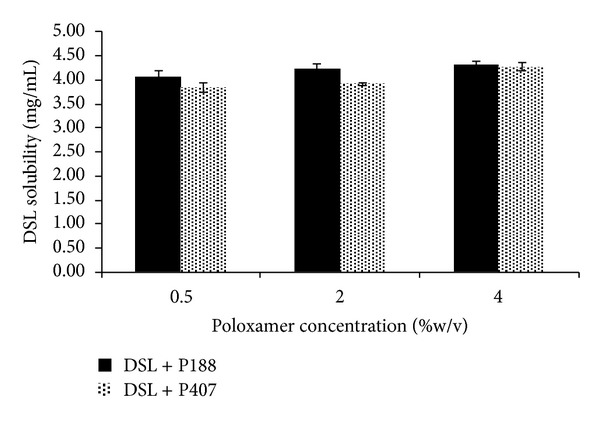
Saturated solubility study of DSL in presence of poloxamer P188 and P407.

Saturated solubility of DSL in PBS was found to be 3.24 ± 0.17 mg/mL. Addition of poloxamer at 0.5% w/v concentration level leads to an increase in solubility of DSL to statistically significant extent but further increase in concentration of poloxamer does not seem to enhance solubility to significant extent (Table 2 and Figure 2). Poloxamers were used as a solubility enhancer because they have bland taste. It is a very important consideration since prepared formulation is intended to be taken by paediatrics. Application of student's paired *t*-test revealed an interesting fact that P188 is superior to P407, and it is in compliance with the literature findings.

### 3.2. Physicochemical Characterization of OS of DSL

**Figure 3:**
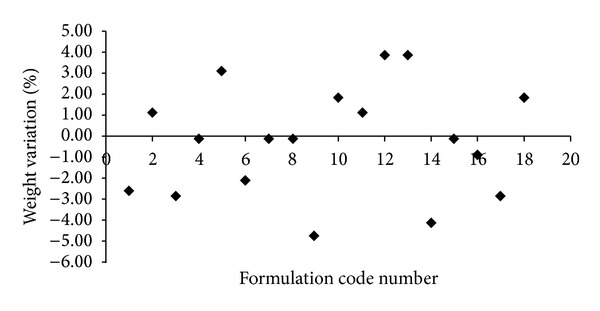
Weight variation analysis of OS of DSL (*n* = 10).

**Figure 4:**
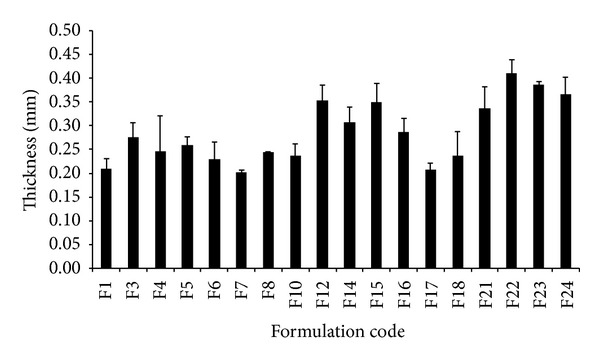
Thickness analysis of OS of DSL (*n* = 10).

As per design of experiment, physicochemical characterization of DSL involves study of three parameters, namely, weight, variation in film, thickness of film, and content uniformity of film. In addition to that it also involves recording of physical observation of film like touch, transparency, flexibility, and presence of air bubbles (blooming) in OS. OS was found to be flexible, transparent, and free from blooming. Data related to weight variation analysis, thickness analysis, and content uniformity analysis is listed in Table 3 and Figures 3 and 4. These are important parameters since they will determine the accuracy of dose administered after intake of dosage form.

Weight variation of all the formulation batches of OS of DSL lies within the range of ±5% (Figure 3). Interbatch variation in thickness was observed to statistically significant extent (*P* < 0.05) as compared to intrabatch variation. It is attributed by the fact that when concentration of plasticizer and polymer varies in formulation, it will change the viscosity of the solution which ultimately affects the spreading behaviour of polymer-plasticizer solution since they thought to exhibit plastic flow. The required shear stress and rate of shear will vary depending upon the viscosity of solution. Hence, viscosity of solution must be measured while making OS by adopting various technologies, especially solvent casting method [19].

### 3.3. Mechanical Properties of Film

Mechanical properties of film were found to be affected by both polymer ratios as well as the concentration of plasticizer incorporated into the film. MDX is known to impart ductility to the formed films and HPMC is known to impart mechanical strength to the film and same results were again revalidated by forming OS of DSL. As the concentration of MDX increased within the formulation, the formed OS was found to have lesser tensile strength and higher percent elongation value. Plasticizer is thought to act by interfering in the polymer-polymer interactions during film formation. The come in between the polymer chains prevent, their ordered arrangement, thereby increase the flexibility of film. Lesser the concentration of plasticizer stiffer will be the film or vice versa [20]. Results of analysis of mechanical properties OS of DSL are reported in Table 4.

Statistical analysis of mechanical properties of OS of DSL shows that plasticizer has pronounced effect of mechanical properties of film [21]. Tukey's HSD was used as a statistical tool to compare the mechanical properties of different OS formulations at 99.99% confidence level (*P* < 0.01). Clearly, tensile strength and percent elongation are inversely proportional and depend upon concentration of plasticizer present in formulation (see results of F2, F12, F17, and F19).

### 3.4. Moisture Uptake

**Figure 5:**
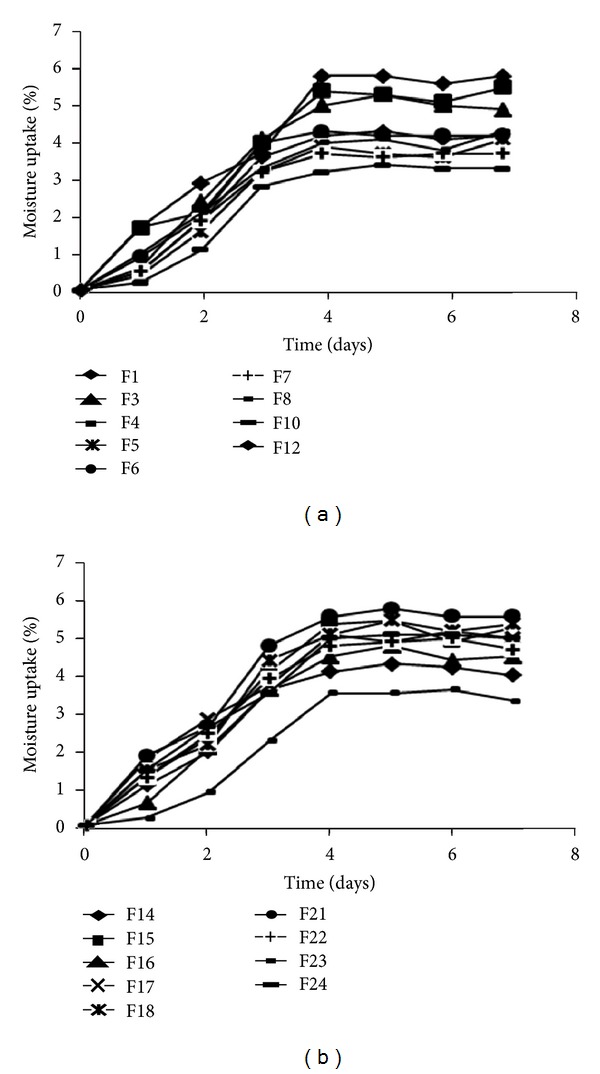
Moisture uptake analysis: (a) formulations containing glycerine and (b) formulations containing PEG 400.

Since fast disintegrating technology relies on the use of hygroscopic excipients it is generally liable for moisture uptake. The extent of moisture uptake depends upon the concentration of hygroscopic excipient present. OS is not an exception to this. MDX with high DE value is known to have hygroscopic characterization [17, 22]. Presence of plasticizer further aids in moisture uptake. Moisture analysis studies guide us about the type of packaging and storage conditions a particular dosage form requires. It was found that as the concentration of MDX and plasticizer increases percent moisture uptake increases. All the formulations were found to attain equilibrium moisture uptake within 4 days of study after that further there was no increase in weight of OS formulations (Figure 5).

### 3.5. *In Vitro* Disintegration Time

An attempt has been made to simulate the physiological conditions while carrying out *in vitro* disintegration test. In simulated conditions, saliva from the salivary glands is secreted at the rate of 1 mL/minute. The volume of saliva that can be hold in buccal pouch is 6 mL only, and diameter of sublingual pouch is mentioned to be 3 to 4 cm. Oral cavity is subjected to minimum agitation. Therefore, in light of above mentioned facts, it is inappropriate to use traditional disintegration apparatus for studying the disintegration time. Hence, a Petri dish of 5 cm in diameter (comparable to diameter of sublingual pouch) containing 10 mL of PBS as disintegration media (comparable to volume of sublingual pouch) maintained at 37°C ± 2°C, occasionally swirled after every 10 seconds (to simulate minimum agitation conditions of oral cavity), was considered as an appropriate method for studying disintegration time. Results of disintegration test are reported in Table 5. Data reveals that presence of high amount of MDX and plasticizer yields statistically significant disintegration time (*P* < 0.05). Tukey's HSD test was used to unfold the effect of type of plasticizer on disintegration time. Glycerine yields lower disintegration time, in statistically significant manner, over PEG 400 (*P* < 0.01).

### 3.6. Surface pH of Film

Alteration in pH of oral cavity is a matter of concern, especially when dosage form is intended to be taken by paediatrics. Minor change in pH of oral cavity can cause irritation which can lead to spitting of dosage form by the child. Average pH of oral cavity varies within the range of 6.4 to 6.8. Prepared film formulations do not cause significant change in pH of oral cavity (Table 5). Hence, designed OS formulations are suitable for oral consumption.

### 3.7. *In Vitro* Dissolution Testing

**Figure 6:**
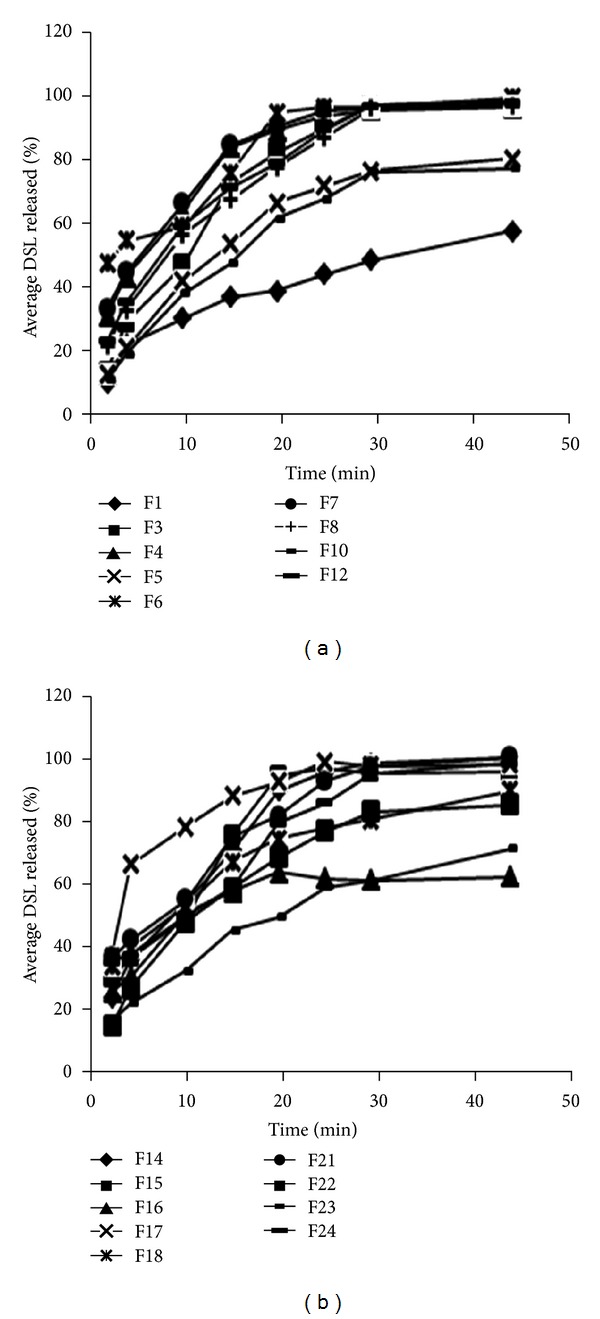
*In vitro* dissolution test of OS loaded with DSL: (a) formulations containing glycerine and (b) formulations containing PEG 400.

Since OS of DSL is a fast disintegrating dosage form, it will release its entire drug content within a short span of time (in minutes). Therefore, release of drug at 4 minutes was considered as a measure for analysis. Formulations containing high amount of MDX and plasticizer (5 : 5 ratio and 25% plasticizer concentration) were found to dissolute at much faster rate as compared to other formulations (Table 5). Tukey's HSD test reveals that formulation containing glycerine as a plasticizer dissolutes at faster pace in comparison to PEG 400 containing formulations (*P* < 0.01). In order to explore the role of MDX in formulation, an additional formulation was also prepared containing only HPMC E5 and 20% plasticizer. This formulation was found to have statistically significant lower dissolution rate. This is because of high solubility of MDX (Figure 6).

### 3.8. Determination of Moisture Content in OS of DSL

Water content of optimized formulations (F2, F12, F17 and F19) was measured by storing the formulation in desiccator as well as in humidity chamber at 60% RH. Increase in moisture content in formulations was measured and was statistically compared using paired student's *t*-test. No significant increase in moisture was observed in case of formulations stored in desiccator, but formulation stored in humidity chamber shows significant increase in moisture level (*P* < 0.01). Highest moisture uptake was shown by formulation containing 25% w/w glycerine along with MDX and HPMC E5 in ratio of 5 : 5. As OS contains hygroscopic excipients, which are liable of moisture uptake, OS should be stored in air tight containers.

### 3.9. Scanning Electron Microscopy (SEM) of Film

**Figure 7:**
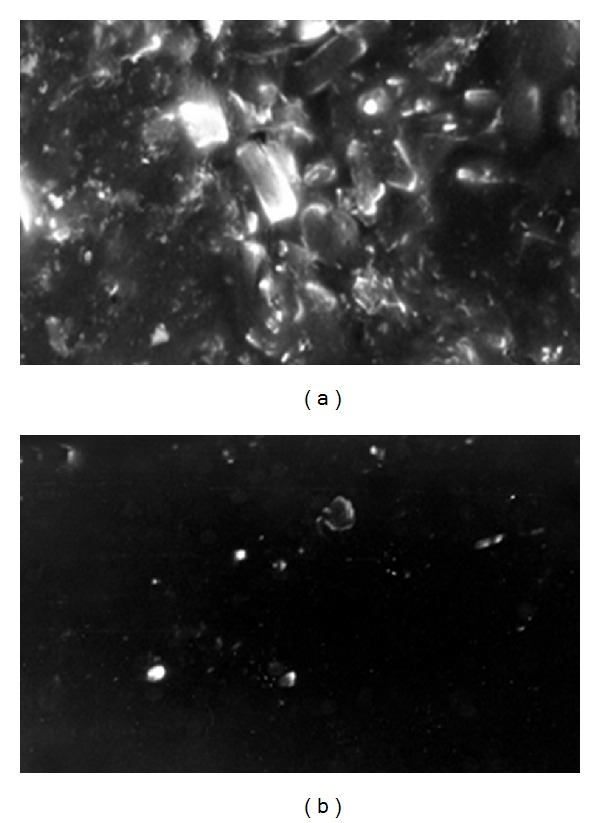
SEM of optimised formulation F12 (a) without poloxamer P188 and (b) with poloxamer P188.

SEM shows that in absence of poloxamer P188, during drying process drug crystallise which can be easily observed in microscope while addition of poloxamer prevents the crystallisation of drug by stabilizing its molecular dispersion in polymer matrix (Figure 7). 

### 3.10. Histological Studies

Optimized formulation was exposed to various portions of freshly excised porcine oral mucosa. Porcine oral mucosa was chosen as a model tissue because of its resemblance to human oral mucosa and thus will provide better simulation of human oral cavity. Histological studies reveal that no structural changes were induced by exposing optimized formulation (F12) to different regions of porcine oral mucosa over a period of 2 hours. 

### 3.11. Accelerated Stability Studies

No statistically significant difference (paired student's *t*-test, *P* < 0.01) were observed in drug content, surface pH, *in vitro* disintegration time, and *in vitro* dissolution time at different time points during 3 months of accelerated stability studies. Bracketing technique was adopted during accelerated stability studies which involves observation of stored samples at accelerated conditions on terminal time points (initial and finial point). Optimized formulation (F12) shows robust results when stored in air tight packing, for example, sealed aluminium pouch or air tight container. 

## 4. Conclusion

The presented work was an attempt to develop a novel OS of DSL for paediatric usage which will circumvent the problems associated with liquid dosage forms usually prescribed to paediatrics during AR. Blend of MDX and HPMC E5 was characterised at different ratio levels in the presence of different types of plasticizer (PEG 400 and glycerol) at variable concentration levels. It was found that the desired characteristic of OS was exhibited by formulation containing glycerine 25% w/w and MDX and HPMC E5 in 5 : 5 ratio. Optimized formulation does not change pH of mouth to significant extent and does not induce any structural changes when it comes in contact with different regions of oral mucosa, but proper storage of OS is a critical factor to be considered. 

## Figures and Tables

**Table 1:** Optimal design of OS of DSL (Design-Expert version 8 software).

Formulation code	Plasticizer	Plasticizer (%)	MDX : HPMC
F1	Glycerine	15.0	3 : 7
F2	Glycerine	20.0	5 : 5
F3	Glycerine	20.0	3 : 7
F4	Glycerine	20.0	5 : 5
F5	Glycerine	20.0	1 : 9
F6	Glycerine	25.0	3 : 7
F7	Glycerine	25.0	1 : 9
F8	Glycerine	15.0	5 : 5
F9	Glycerine	25.0	3 : 7
F10	Glycerine	15.0	1 : 9
F11	Glycerine	20.0	1 : 9
F12	Glycerine	25.0	5 : 5
F13	Glycerine	15.0	3 : 7
F14	PEG 400	25.0	1 : 9
F15	PEG 400	25.0	3 : 7
F16	PEG 400	15.0	3 : 7
F17	PEG 400	20.0	5 : 5
F18	PEG 400	20.0	3 : 7
F19	PEG 400	25.0	5 : 5
F20	PEG 400	15.0	1 : 9
F21	PEG 400	25.0	5 : 5
F22	PEG 400	20.0	1 : 9
F23	PEG 400	15.0	1 : 9
F24	PEG 400	15.0	5 : 5

**Table 2:** Saturated solubility studies of DSL (alone and in presence of poloxamers).

Composition	Solubility in PBS (mg/mL)				
	N1	N2	N3	Mean	SD
DSL	3.21	3.43	3.09	3.24	0.17
DSL + P188 (0.5% w/v)	3.92	4.04	4.21	4.06	0.15
DSL + P188 (2.0% w/v)	4.11	4.26	4.3	4.22	0.10
DSL + P188 (4.0% w/v)	4.24	4.37	4.33	4.31	0.07
DSL + P407 (0.5% w/v)	3.74	3.96	3.82	3.84	0.11
DSL + P407 (2.0% w/v)	3.89	3.95	3.91	3.92	0.03
DSL + P407 (4.0% w/v)	4.2	4.27	4.35	4.27	0.08

N1, N2, and N3: replicate measurements; SD: Standard Deviation.

**Table 3:** Physicochemical characterization of OS of DSL (*n* = 10).

Formulation code	Content (%)	Weight variation (%)	Thickness (mm)
F1	97.23	−2.83	0.21 ± 0.02
F3	98.88	1.16	0.28 ± 0.03
F4	99.2	−2.83	0.25 ± 0.07
F5	99.92	−0.17	0.26 ± 0.02
F6	100.13	3.16	0.23 ± 0.04
F7	101.34	−2.16	0.202 ± 0.00
F8	103.02	−0.17	0.24 ± 0.00
F10	99.4	−0.17	0.24 ± 0.03
F12	97.11	−4.83	0.35 ± 0.03
F14	97.43	1.83	0.31 ± 0.03
F15	99.92	1.16	0.35 ± 0.04
F16	100.45	3.83	0.29 ± 0.03
F17	104.32	3.83	0.21 ± 0.02
F18	102.09	−4.16	0.24 ± 0.05
F21	99.89	−0.17	0.34 ± 0.05
F22	103.78	−0.83	0.41 ± 0.03
F23	101.22	−2.83	0.39 ± 0.01
F24	100.32	1.83	0.37 ± 0.04

**Table 4:** Mechanical properties of OS of DSL.

Formulation code	Tensile strength (MPa)	Elongation (%)
F1	13.1	22.9
F3	12.6	27.8
F4	9.0	45.2
F5	11.5	65.4
F6	2.7	69.9
F7	11.4	78.9
F8	30.2	42.5
F10	12.1	37.1
F12	2.5	88.2
F14	11.8	63.8
F15	10.2	52.3
F16	25.4	34.1
F17	8.8	72.3
F18	12.4	38.3
F21	3.1	78.8
F22	14.8	63.5
F23	24.3	39.7
F24	11.2	75.4

**Table 5:** *In vitro* disintegration test, surface pH test, and *in vitro* dissolution test of OS loaded with DSL.

Formulation code	Disintegration time (s)	Surface pH	Dissolution (%) within 4 minutes
F1	5	6.7	24.45
F3	9	6.7	31.24
F4	12	6.7	45.75
F5	12	6.7	23.26
F6	15	6.8	57.34
F7	20	6.6	47.32
F8	16	6.8	35.37
F10	24	6.5	21.2
F12	9	6.7	37.66
F14	21	6.8	37.82
F15	13	6.8	38.34
F16	17	6.8	32.11
F17	14	6.7	67.83
F18	15	6.8	41.34
F21	11	6.8	43.91
F22	17	6.6	28.6
F23	21	6.7	23.78
F24	17	6.7	39.21
